# Rare case of intracerebral hemorrhage in anaphylactic shock following administration of intramuscular adrenaline: A case report

**DOI:** 10.1002/ccr3.6534

**Published:** 2022-11-15

**Authors:** Shunki Yamamoto, Takashi Hongo, Tomokazu Tamura, Tetsuya Yumoto, Hiromichi Naito, Atsunori Nakao

**Affiliations:** ^1^ Department of Emergency, Critical Care, and Disaster Medicine Okayama University Graduate School of Medicine, Dentistry, and Pharmaceutical Sciences Okayama Japan; ^2^ Emergency Department Okayama Saiseikai General Hospital Okayama Japan

**Keywords:** adrenaline, anaphylactic shock, dialysis, intracranial hemorrhage

## Abstract

Intracerebral hemorrhage should be considered as a possible adverse event in patients with anaphylactic shock who are treated with adrenaline administration, especially in those at high risk of serious bleeding events.

## Abstract 2

Although prompt intramuscular injection of adrenaline is the first‐line treatment for anaphylactic shock, physicians should recognize that adrenaline injection can lead to intracerebral hemorrhage, especially in high bleeding risk patients.

## 1 INTRODUCTION

Anaphylaxis with rapidly life‐threatening systemic hypersensitivity reactions occurring after being triggered by inflammatory mediators can lead to fatal airway obstruction and shock.^1^, ^2^ Appropriate acute management of anaphylactic shock involves early administration of intramuscular adrenaline, which has a significant impact on favorable outcomes.^3^, ^4^ However, previous published reports have rarely shown the incidence of intracranial hemorrhage following anaphylactic shock treated with adrenaline injection.^5^, ^6^ Furthermore, intracerebral hemorrhage can occur after adrenaline administration via any route, including in patients undergoing therapeutic upper‐gastrointestinal endoscopy and those with asthma.^7^, ^8^ Although a definitive pathogenesis of these adverse events has not been elucidated, these serious adverse events can be fatal and require further intervention. In addition, the number of elderly people with multiple comorbidities and taking many medications who are at high risk for hemorrhage may increase every year.^9^

Sharing our experience may help clinicians recognize cerebrovascular complications after injection of adrenaline in bleeding risk patients with anaphylactic shock; adrenaline injection is the first‐line management for these patients.

## 2 CLINICAL CASE

A 75‐year‐old‐female patient who undergo hemodialysis treatment for chronic kidney disease for over 4 years visited her family physician's clinic complaining of fever. She denied any personal history of drug allergy or anaphylaxis and no allergic reactions had previously occurred. She had a history of hypertension, dyslipidemia, and arteriosclerosis obliterans treated with axillary‐femoral bypass, for which she was prescribed aspirin 100 mg per day. She was a former smoker with a quarter pack of cigarettes per day for 50 years.

Empirically, she was given intravenous antibiotics (1 g dose of cefoperazone‐sulbactam) for a suspected bacterial infection 3 h after initiation of hemodialysis. A few minutes later, she proceeded to develop periorbital edema, dyspnea, and altered consciousness, and her blood pressure fell to 54/30 mmHg. Her family physician diagnosed her with anaphylactic shock caused by the antibiotic and intramuscularly administered 0.3 mg dose of adrenaline (1:1000 dilution) twice every 5 min, as well as 5 mg dose of chlorpheniramine maleic acid, 20 mg dose of famotidine, and 125 mg dose of methylprednisolone. A few minutes after receiving adrenaline, the patient developed tachycardia with a pulse rate of 140 beats/min and hypertension with a blood pressure of 208/88 mmHg. Thirty minutes after onset upon arrival of emergency medical services personnel at the scene, her blood pressure fell to 94/44 mmHg. She was admitted to our institution for further treatment 1 h after the incident.

En route to the emergency department, she appeared pale. She had a Glasgow Coma Scale (GCS) score of 12 (E2V4M6), respiratory rate of 20 breaths/min, arterial oxygen saturation of 100% with oxygen delivered through a face mask (3 L/min), pulse rate of 110 beats/min, blood pressure of 104/78 mmHg, and body temperature of 37.1°C. Neurological examination revealed no focal deficits except for altered mental status, presumably attributed to anaphylactic shock.

**FIGURE 1:**
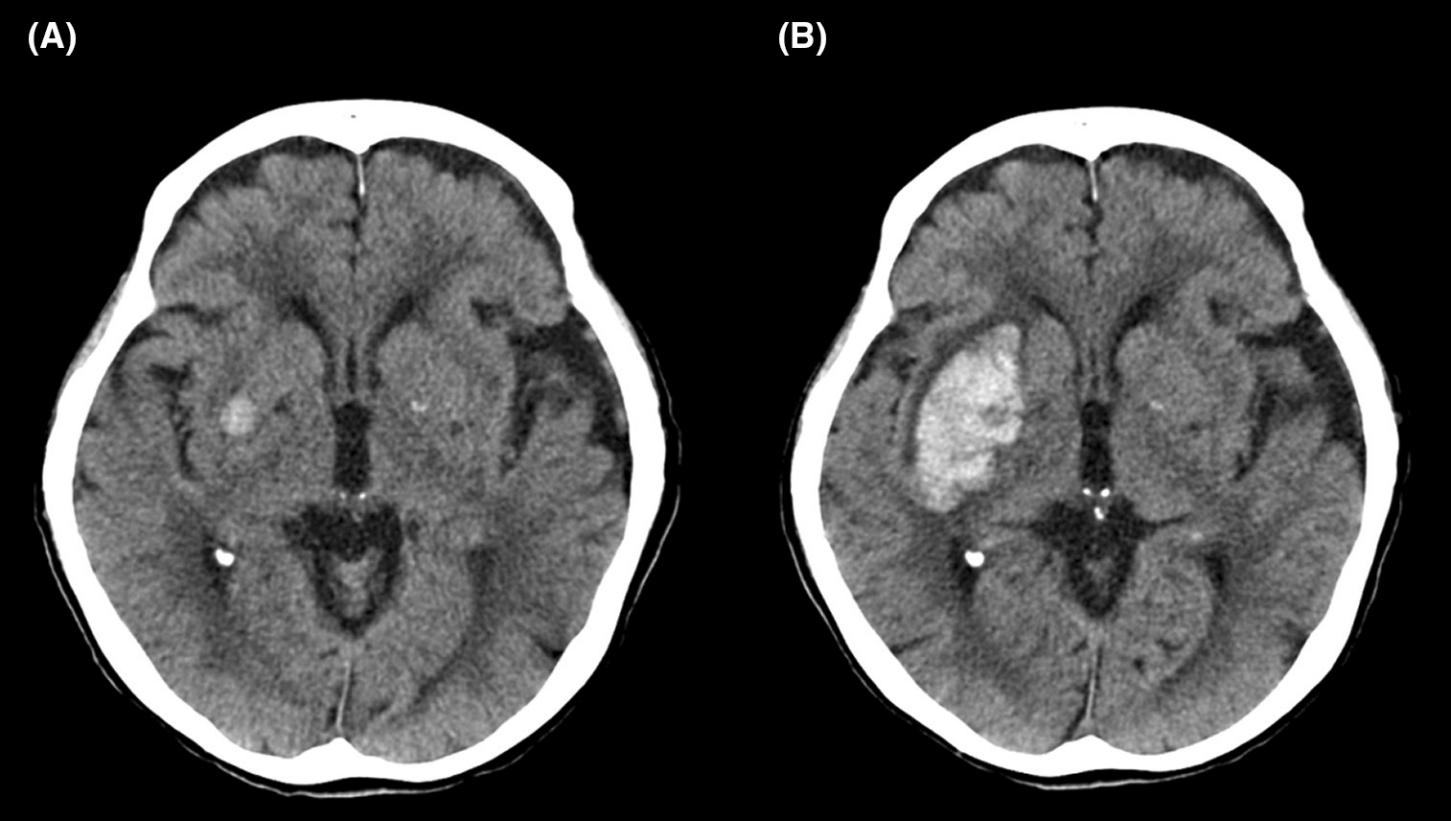
Computed tomography scan of the patient's head. A: An intracranial hemorrhage measuring 9 × 9 × 5 mm was revealed in the right basal ganglia on admission. B: The intracranial hemorrhage increased to 52 × 35 × 30 mm 3 h after admission

Laboratory test results demonstrated chronic kidney disease as indicated by urea nitrogen levels of 17 mg/dL, elevated creatinine levels of 4.86 mg/dL, thrombocytopenia (blood platelets cell count of 9.0 × 10^4^ cells/L), normal prolonged prothrombin time (11.5 s), and activated partial thromboplastin time (43.1 s). Blood culture test did not identify any bacteria. A head computed tomography (CT) scan was conducted to determine the causes of altered consciousness other than anaphylactic shock. The CT scan revealed a hemorrhagic lesion measuring 9 × 9 × 5 mm in the right basal ganglia (Figure 1A). We conducted conservative treatment using nicardipine hydrochloride to promptly stabilize her systolic blood pressure to under 140 mmHg, as well as tranexamic acid to prevent a second bleeding and hematoma enlargement. Neurological examination showed the patient failed to achieve complete clarity of consciousness; she had a GCS score of 11 (E2V3M6) and her left upper and lower limbs were paralyzed without sensory deficits 4 h after onset. We performed a follow‐up head CT scan, which showed that the intracerebral hemorrhage had increased in size to 52 × 35 × 30 mm (Figure 1B). During the first few days, the patient presented no additional neurological deterioration without further expansion of the intracerebral hemorrhage.

The patient was transferred to the local rehabilitation hospital 28 days after admission with weakness of the left upper and lower limbs, which affected her daily life, and a modified Rankin Scale score of 5.

## 3 DISCUSSION

We herein report a 75‐year‐old female patient on dialysis with serious sequelae caused by intracerebral hemorrhage following anaphylactic shock treated with intramuscular administration of adrenaline.

Leukopenia, non‐occlusive mesenteric ischemia, ischemic stroke, and intracerebral hemorrhage are adverse events after anaphylactic shock that have previously been reported in the medical literature.^5^, ^10^, ^11^, ^12^, ^13^

Although other etiologies cannot be excluded, we assume that acute hypertensive attack induced by adrenaline and dialysis with a high risk of hemorrhage may be associated with the pathogenesis of intracerebral hemorrhage, in our case at least partially. Indeed, during anaphylactic shock, our patient's cerebral blood flow decreased more than what could be anticipated based on the blood pressure observed in animal experiments.^14^ Therefore, the pathogenesis of intracerebral hemorrhage may have etiologies other than anaphylaxis. Similar to our case, previous reports have hypothesized that intracerebral hemorrhage after anaphylactic shock can result from elevated blood pressure induced by adrenaline administration when there is no evidence of vascular abnormality or intracerebral tumor.^5^, ^6^

Adrenaline is one of the most commonly used medications to manage conditions such as cardiac arrest, asthma, septic shock, and anaphylactic shock, but serious adverse events can occur, including arrhythmias, lactic acidosis, pulmonary edema, and cerebrovascular disease.^6^, ^15^, ^16^, ^17^ Intracerebral hemorrhage after administration of adrenaline via different routes, including intravenous and intramuscular administration and inhalation, has previously been described.^5^, ^8^, ^18^ Adrenaline can lead to stimulation of all α and β adrenergic receptors, eliciting short‐term systolic hypertension, and suppress inflammatory mediators released from mast cells and basophils.^19^ Adrenaline influences a significant dysfunction of cerebral autoregulation and the blood brain barrier in animal experiments.^20^ These pharmacological mechanisms may have contributed to intracerebral hemorrhage.

Remarkably, other multiple factors may also have induced intracerebral hemorrhage and development of hematoma expansion in the present case. First, higher age and smoking history or underlying diseases including hypertension, dyslipidemia, and chronic kidney disease could precipitate the intracerebral hemorrhage.^21^, ^22^ Second, aspirin use might be associated with intracerebral hemorrhage and its enlargement.^23^ Third, dialysis could be responsible for intracerebral hemorrhage due to (1) altered platelet‐vessel wall interactions and platelet dysfunction, and (2) anticoagulant use during dialysis.^24^ Physicians are warranted to carefully observe the anaphylactic shock patients after adrenaline injection in terms of intracranial hemorrhage, since the number of elderly people with multiple high bleeding comorbidities tends to increase every year.^9^

## 4 CONCLUSION

Intracranial hemorrhage following anaphylactic shock treated with intramuscular administration of adrenaline should be recognized as an adverse event, especially in high bleeding risk patients.

## AUTHOR CONTRIBUTIONS

Shunki Yamamoto, Takashi Hongo, and Atsunori Nakao conceptualized the study Shunki Yamamoto, Takashi Hongo, Tomokazu Tamura, and Atsunori Nakao performed data curation. Shunki Yamamoto, Takashi Hongo, Tetsuya Yumoto, Hiromichi Naito, and Atsunori Nakao investigated the study. Shunki Yamamoto and Takashi Hongo prepared original draft. Takashi Hongo, Tetsuya Yumoto, Hiromichi Naito, and Atsunori Nakao reviewed and edited the manuscript.

## CONFLICT OF INTERESTS

The authors declare that they have no competing interests.

## ETHICAL APPROVAL

Written informed consent for participation was obtained.

## CONSENT

Written informed consent was obtained from the patient for the publication of this case report and the accompanying images. A copy of the consent form is available for review by the Editor‐in‐Chief of this journal.

## Data Availability

The datasets used and/or analyzed during the current study are available from the corresponding author upon reasonable request.
